# Case Report: BKV-specific T cells: a fast, safe and potentially effective treatment option for refractory BKV infections in pediatric patients after allogeneic stem cell transplantation

**DOI:** 10.3389/fped.2026.1732453

**Published:** 2026-05-07

**Authors:** Sven Oberwegner, Steffen A. Hettler, Luca Hensen, Amadeus T. Heinz, Christiane Braun, Michaela Döring, Peter Lang

**Affiliations:** 1 Department of Pediatrics I, Hematology and Oncology, University Hospital Tübingen, Tübingen, Germany; 2 Department of Internal Medicine II, Hematology, Oncology, Clinical Immunology, and Rheumatology, University Hospital Tübingen, Tübingen, Germany; 3 Stuttgart Cancer Center, Zentrum für Kinder-, Jugend- und Frauenmedizin (Olgahospital), Pädiatrie 5 (Pädiatrische Onkologie, Hämatologie, Immunologie), Klinikum der Landeshauptstadt Stuttgart, Stuttgart, Germany

**Keywords:** adoptive T cell therapy, BK virus, BKV-specific T cells, CliniMACS Prodigy, hematopoetic stem cell transplantation

## Abstract

BK virus reactivation is frequently observed in patients following allogeneic stem cell transplantation and can cause nephropathy and hemorrhagic cystitis. Current treatments are mainly supportive, and antiviral therapy with cidofovir lacks clear evidence of efficacy. Therefore, novel therapeutic strategies are needed. Since adoptive transfer of virus-specific T cells has proven effective for other viral infections, this case series with four cases of post-transplant severe BKV infections evaluated the feasibility, safety, and efficacy of BKV-specific T cells in patients with refractory BKV infection using the CliniMACS Prodigy system. Three of the four patients demonstrated a reduction in BKV viral load following adoptive T-cell transfer, with detectable *in vivo* expansion of BKV-specific T cells in these individuals. However, complete viral clearance was not achieved in all cases. One patient with extensive immunosuppression showed minimal viral response and no detectable BKV-specific T cells after infusion. Treatment was well tolerated, with no infusion-related toxicities and no severe *de novo* graft-versus-host disease observed. Overall, this case series suggests that rapidly manufactured BKV-specific T cells produced via the CliniMACS Prodigy system represent a feasible, safe, and potentially effective treatment option for refractory BKV viremia after stem cell transplantation. Early administration and limited concurrent immunosuppression may improve therapeutic outcomes. Further studies are needed to confirm these findings.

## Introduction

BK virus (BKV), which is usually acquired in infancy (1), remains latent in numerous cell types of the body (2, 3). In up to 81% of patients who receive allogeneic hematopoietic stem cell transplantation (HSCT), virus reactivation in blood or urine is observed (4). Hemorrhagic cystitis (HC), the most common symptom in those patients, is painful, prolongs the in-hospital stay and has been associated with an increased mortality rate (5, 6). Current treatment strategies comprise symptomatic approaches like intravenous hydration, continuous bladder irrigation, application of tranexamic acid, ciprofloxacin and estrogen or the use of the antiviral agent cidofovir. However, to date, clear evidence of clinical efficacy of cidofovir against BKV-induced disease is still missing (7), and new therapeutic approaches are needed. Several authors suggest the use of BKV-specific T cells in case of refractory BKV infections (8, 9). Since the adoptive transfer of virus-specific T cells has been shown to be effective in ADV-, CMV- and EBV-infections (10), with this case series we report our experiences with BKV-specific T cells in patients with refractory BKV infection.

## Methods

Virus-specific T cells were produced in a cytokine capture system (CCS) as described before in a central GMP laboratory at the University Children's Hospital Tübingen (11). Potential donors were screened for BKV-specific T cell responses, and only donors with demonstrable reactivity against BKV antigens were selected for manufacturing. If no BKV-specific T cells were detectable in the original stem cell donor, a third-party donor (parent) with these T cells was used. In brief, with an unstimulated apheresis out of donor blood 1 × 10^9^ white bloods cells were collected. T cells were stimulated for four hours with MACS GMP PepTivators BKV VP1 and BKV-LT (170-076-138 & 170-076-139, Miltenyi Biotec, Bergisch Gladbach, Germany) under full GMP conditions in an automated CliniMACS Prodigy system (Miltenyi Biotec). IFN-γ-secreting T cells were labeled with CliniMACS catch reagent and microbeads (200-070-111, Miltenyi Biotec) and magnetically enriched. Cells were either applied fresh after production or frozen in 10% dimethyl sulfoxide (DMSO) until application. Release criteria included i) purity >20% CD3^+^ cells, ii) sterility and iii) viability 10%–100% with a dose of up to 1 × 10^5^ T cells/kg body weight for HLA-matched donors and up to 2.5 × 10^4^ T cells/kg body weight for HLA-mismatched haploidentical donors (parent).

In case of concurrent viral reactivations (e.g., CMV, EBV, or ADV) multi-virus–specific T cells (multi-VST) rather than BKV-only VST were produced by stimulation with additional virus-specific peptide pools targeting the respective co-reactivating viruses. Here too were only donors selected who were screened before the apheresis for the desired virus-specific T cell response to ensure specificity. Post-manufacturing BKV-specificity testing of the final product was not performed. However, based on our prior manufacturing experience using the same stimulation strategy, we have consistently observed preservation of antigen-specific responses, including BKV specificity, in multi-VST products generated from pre-screened donors.

Presence of BKV specific T cells (CD4^+^ and CD8^+^) after VST application was assessed using an IFN-γ assay following stimulation with BKV-derived peptide pools and flow cytometry.

## Results

**Figure 1:**
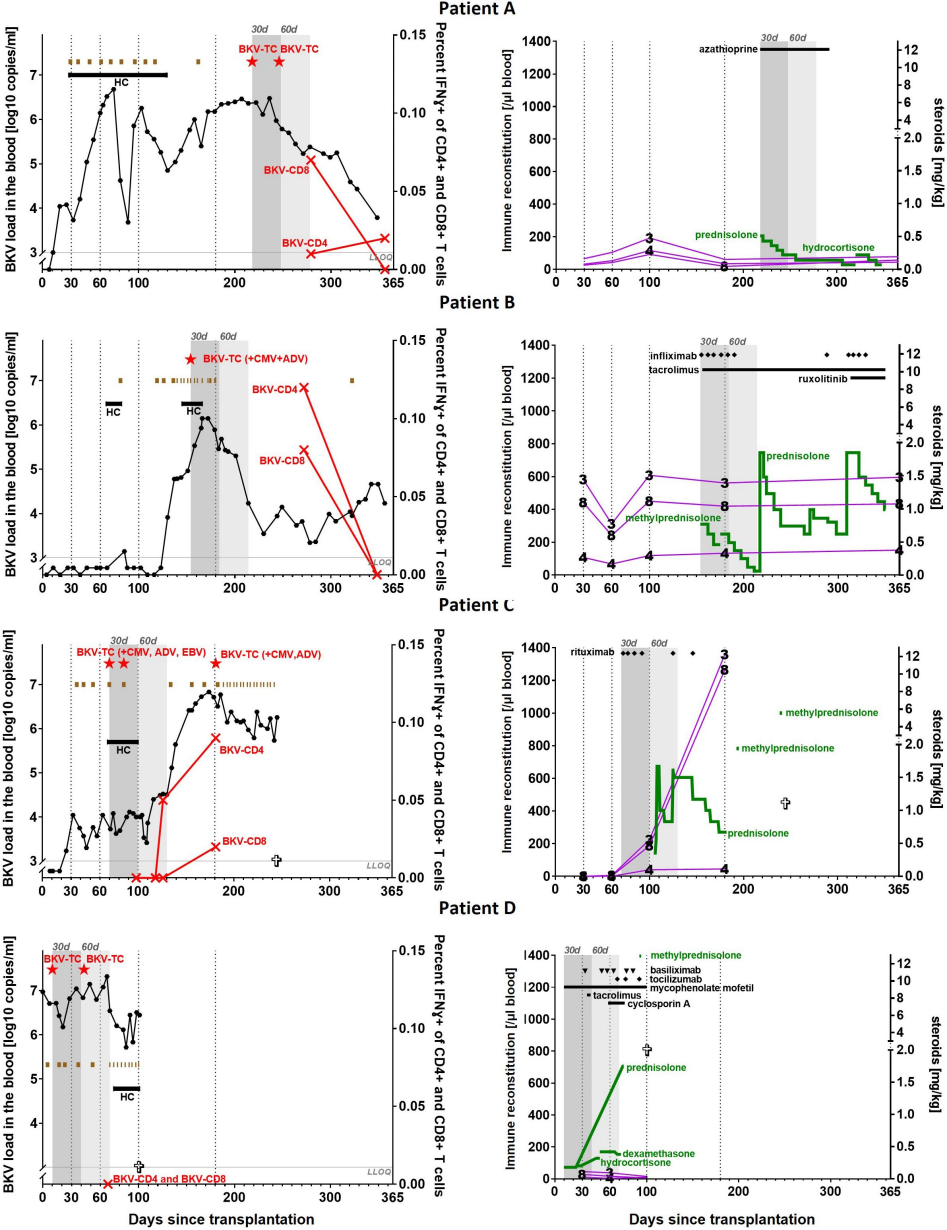
Clinical course of the four reported patients (A, B, C, D) with treatment-refractory BK viremia. Each row represents one patient. **First Column**: Course of BKV load in the blood with a lower limit of quantification (LLOQ) of 1000 copies/ml blood, duration of hemorrhagic cystits (HC), timepoint of death (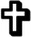) and treatment overview with the following options: cidofovir (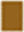), brincidofovir (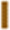), BKV-specific T cells (BKV**-**TC;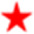) and multispecific T cells (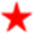) and detectable BKV-specific T cells (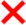). The first 30/60 days after administration are in darker/brighter grey highlighted. **Second Column**: Immune reconstitution (in purple) with the cell counts of CD3, CD4 and CD8 positive cells, assessed by flow cytometry. Different agents of immunosuppression (in black), which were used after administration of BKV-specific T cells. The dosage (by body weight) of the steroids can be found on the right y-axis (in green).

34 allogeneic stem cell transplantations were performed in our center in 2017. In this cohort, a total of four patients were treated with BKV-specific T cells. Indication for transplantation in those four patients were myelodysplastic syndrome (Patient A, C), thalassemia major (Patient B) and hemophagocytic lymphohistiocytosis (Patient D). They received grafts from matched unrelated (A, B, C) or mismatched related haploidentical donors (D) after myeloablative conditioning regimens. Serotherapy was carried out with Thymoglobulin (A, B, C) and Anti-T-lymphocyte globulin (ATLG) (Grafalon) (D). All patients received unmanipulated bone marrow. BKV load was monitored at least once a week. The detailed clinical course of BKV infection of each patient and its treatment is shown in Figure 1. We detected BKV DNA in the urine 4–9 days post-transplant and in the blood within 8–56 days post-transplant in all four patients. Clinical symptoms of hemorrhagic cystitis occurred within 28–74 days post-transplant. The mildest symptoms had patient C with macroscopic hematuria without clots. The other three patients suffered under macroscopic hematuria with clots. Neither required instrumentation for clot evacuation. Patients received treatment with intravenous hydration, ciprofloxacin, tranexamic acid, dose reduction of the immunosuppression. Intravenous cidofovir treatment was initiated in all patients, but wasn’t able to clear the virus from blood or urine.

Due to poor immune recovery in all patients with no detectable BKV-specific T cells (only tested in patient C and D), adoptive transfer of BKV-specific or multi-specific T cells from a third party (haploidentical parent, A, B) or original stem cell donor (C, D) was initiated. Patients received 5.09–32.5 × 10^3^ BKV-specific T cells/kg body weight immediately after the 48 h separation procedure. For patients A, C and D additional VST were cryopreserved and a second transfer with the same amount of VST was done. In the case of patient C a third transfer was performed from a second separate cultivation with VST from the same donor. Patients A and D received only BKV-specific T cells, patient B received multispecific VST against ADV, CMV and BKV, and patient C received multispecific VST against ADV, CMV, EBV and BKV.

All four patients received several different immunosuppressive agents due to atypical hemolytic uremic syndrome (A), post-transplant lymphoproliferative disorder (B, C) and persistent or resolving GvHD (B, C) and hemophagocytic lymphohistiocytosis (D) during and after administration of VST (see Table 1). Three patients (B, C, D) suffered from a coexisting adenoviral infection, which was treated with brincidofovir on a compassionate use basis. In all three patients ADV was detected in stool and blood. Since routine urine testing for ADV is not performed in our center, we have no data on the ADV urine status of our patients.

**Table 1:** Overview on patient and transplant characteristics, transfer of BKV-specific T cells, the course of BKV infection after T-cell transfer and the occurrence of graft-vs.-host disease.

Characteristics		Patient A	Patient B	Patient C	Patient D
Patient and transplant characteristics	Age (years)	11	3	4	4
	Sex	Male	Male	Male	Female
	Diagnosis	MDS	Beta thalassemia	MDS	HLH
	Donor	MUD	MUD	MUD	Haplo
	No. mismatch	1	0	0	5
	Graft	BM	BM	BM	BM
	Conditioning	Fludarabine Treosulfan Thymoglobulin	Fludarabine Busulfan Thiotepa Thymoglobulin	Fludarabine Thiotepa Thymoglobulin	Fludarabine Thiotepa Treosulfan PT-Cy ATLG
	GvHD prophylaxis	CSA MTX MMF	CSA MTX	CSA MTX	MMF Tacrolimus
	Cause of death	n.a.	n.a.	Abdominal PTLD leading to intestinal obstruction and consequently cardiovascular failure	Sepsis leading to ARDS and cardiovascular failure
Transfer of BKV-specific T cells	Number of administrations	2	1	3	2
	Day post-HSCT	d+218 d+246	d+154	d+70 d+85 d+181	d+10 d+43
	CD3+ cells/kg	5,090	20,160	32,470 (d+70, d+85) 10,760 (d+181)	13,300
	Kind of CD3 administered	BKV-T cells	BKV-T cells ADV-T cells CMV-T cells	BKV-T cells ADV-T cells CMV-T cells EBV-T cells*	BKV-T cells
	Origin of CD3	Third party (parent)	Third party (parent)	Donor-derived	Donor-derived
	Additional antiviral treatment post-immunotherapy (first 60 days)	No	BCV	CDV	CDV
	Immunosuppression post-immunotherapy (first 60 days)	Steroids Azathioprin	Steroids Tacrolimus Infliximab	Steroids Rituximab	Steroids Basiliximab Tocilizumab MMF CSA Tacrolimus
	*In vivo* expansion of BKV specific T-cells post-immunotherapy	Yes	Yes	Yes	No
	Peak levels of BKV-specific T cells	0.02% (CD4^+^) 0.07% (CD8^+^)	0.12% (CD4^+^) 0.08. % (CD8^+^)	0.09% (CD4^+^) 0.02% (CD8^+^)	n.a.
BKV infection at start of immunotherapy	BKV copies/ml blood	2,300,000	340,000	11,000	5,100,000
BKV infection 30 days post-immunotherapy	Change of BKV copies/ml blood	↓0.57 log	↑0.5 log	↓0.04 log	↑0.13 log
	BKV positivity in urine	No measurement	Urine positive	Urine positive	Urine positive
BKV infection 60 days post-immunotherapy	Change of BKV copies/ml blood	↓0.98 log	↓0.73 log	↑0.46 log	↓0.16 log
	BKV positivity in urine	No measurement	Urine positive	No measurement	Urine positive
Occurrence of GvHD in the first 60 days after first transfer of BKV-specific T cells		No *de novo* GvHD	No *de novo* GvHD *(preexisting intestinal GvHD worsened on day 60 after transfer)*	*De novo* GvHD *(skin II°,* *development on day 37 after first transfer)*	No *de novo* GvHD

ADV, adenovirus; ARDS, acute respiratory distress syndrome; ATLG, anti-T-lymphocyte globulin; BCV, brincidofovir; BM, bone marrow; BKV, BK virus; CDV, cidofovir; CMV, cytomegalovirus; CSA, cyclosporin A; EBV, epstein-barr virus; GvHD, graft-versus-host disease; HLH, hemophagocytic lymphohistiocytosis; HSCT, hematopoietic stem cell transplantation; MDS, myelodysplastic syndrome; MMF, mycophenolate mofetil; MTX, methotrexate, MUD, matched unrelated donor; n.a., not applicable; No., number; PT-Cy, post-transplant cyclophosphamide; PTLD, post-transplant lymphoproliferative disease.

* Only administered at the first and second administration.

As shown in Figure 1, within of two months after the first administration of VST, patient A showed a 0.98-log reduction, while patient B presented a 0.73-log reduction of viral load in the blood. However, both patients could not clear the viremia and stayed positive at a low level for over one year after administration. Patient C cleared his hemorrhagic cystitis in the first month after administration, but did not show a reduction of the viral load within the first two months. Patient D showed the highest viral load in the blood prior to treatment. The administration of BKV-specific T cells was only 10 days post-transplant. While the viral load was reduced by 0.16-log on day 60 after administration, we did not detect any BKV-specific T cells in the blood. Until death, patient D was not able to clear viremia or hemorrhagic cystitis.

To assess if viral clearance was related to adoptive T cell transfer, we assessed the frequency of VST after transfer with an IFN-γ capture assay. As shown in Figure 1, BKV-specific T cells were detectable in 3 out of 4 patients (A, B and C) while the test result of patient D remained negative. In patient A–C, BKV-specific CD4 positive T cells peaked at 0.02%, 0.12% and 0.09% of all CD4 + cells, while CD8 positive T cells peaked at 0.07%, 0.08% and 0.02% of CD8+ T cells. In patient C an increase in BKV-specific CD4+ and CD8+ positive T cells over time was noted. Simultaneously we saw an increase of the whole CD4+ and CD8 positive T cell population, as shown in Figure 1.

A summarization of details on patient and transplant characteristics, transfer of BKV-specific T cells, the course of BKV infection after T-cell transfer and the occurrence of Graft-versus-host disease (GvHD) in the observation period can be found in Table 1.

## Discussion

In this case series we report our findings using BKV-specific T cells for treatment of refractory BKV infection. Transfer of BKV-specific T cells is considered successful, when viral load decreases and *in vivo* expansion can be detected.

We could demonstrate that the administration of BKV-specific T cells can result in significant reduction of the BKV load in the blood. A low viral load is important for the long-term kidney function and the risk of developing a HC. In a study with 136 patients a BKV load in plasma of ≥10,000 copies/mL was associated with a mean decline in estimated glomerular filtration rate (GFR) of 30.6 mL/min/1.73 m^2^ by one year after HSCT (12). Even asymptomatic patients with BKV viremia have a significant lesser GFR two years after HSCT than patients without BKV viremia as a study by Laskin et al. showed (13). Cesaro et al. found that a BKV load of 1,000 copies/mL in plasma was significantly associated with the development of a HC (6). In accordance with our results, previous phase 2 studies have also demonstrated that the administration of BKV-specific T cells can result in a reduction of viral load in patients with BKV infection. Tzannou et al. found in patients with BKV viruria a 96% decrease of viral load by week 12 postinfusion (14). Nelson et al. found in patients with BKV viremia an overall response rate of 75% (treated with donor-derived BKV-specific T cells) and 95% (treated with third party T cells) (15). This corresponds with our observations, which showed that both patients with a significant reduction of BK viremia were treated with third party T cells. Clinical recovery temporally coincided with the decline in BK viral load following VST administration in the patients who survived the first year post-HSCT. It is biologically plausible that improved viral control contributed to the amelioration of HC symptoms. However, given the observational nature of this study and the lack of a control group, a direct causal relationship cannot be definitively established.

We were able to detect BKV-specific T cells in 3 out of 4 patients. This number is comparable to a study of Koldehoff et al., who could detect BKV specific T cells in 6 out of 7 patients (9). It has to be mentioned that the test results cannot differentiate between transferred and *de novo* synthetized BKV-specific T cells, which can be detected as early as 35 days after HSCT (9). In several studies, researchers observed that increasing BK virus-specific immunity was associated with a decrease in viral load (9, 15). We cannot provide additional data to support this observation, as no more than two measurements were performed per patient.

Patients will probably benefit from timely treatment with BK virus-specific T cells after detection of BK viremia. Therefore, the manufacturing process must be short. With our approach BKV specific T cells can be produced within two days in contrast to the approach of expanding VST prior to application, which can take several weeks (15, 16). As T cell responses are HLA-dependent, individuals with rare HLA alleles might not be covered by such off-the-shelf products, leaving vulnerable populations at risk. The advantage of direct *ex vivo* enrichment is that it has little impact on T cell clonality or T cell phenotypes in contrast to *in vivo* expansion which can alter both (17, 18). We assume *in vivo* expansion in the recipient will result in a more natural expansion and in more robust and sustainable responses. However, further studies for validation are required.

The administration of third party and donor-derived BKV-specific T cells was safe. We did not observe any infusion-related toxicities. Moreover, no *de novo* GVHD other than skin GvHD II° occurred. These results reflect those of Olson et al., who also didn’t find any *de novo* GvHD III° or IV° following infusion (16).

Two of the four patients did not have an optimal response. One reason for poor performance might be the concurrent use of immunosuppressive agents. Patient D had nine different immunosuppressive agents and was the only patient in which we were not able to detect any BKV-specific T cells after administration. This suggests that a high amount of immunosuppression lowers the response rates in the usage of BKV-specific T cells.

In summary, we have shown that the use of CliniMACS Prodigy produced BKV-specific T cells could be a fast, safe and practical treatment option for refractory BK viremia. Early administration and limited concurrent immunosuppression may improve therapeutic outcomes. The effectiveness needs to be validated in larger cohorts.

## Data Availability

The raw data supporting the conclusions of this article will be made available upon reasonable request from the corresponding author.
